# Increased Expression of Resistin in MicroRNA-155-Deficient White Adipose Tissues May Be a Possible Driver of Metabolically Healthy Obesity Transition to Classical Obesity

**DOI:** 10.3389/fphys.2018.01297

**Published:** 2018-10-12

**Authors:** Candice Johnson, Charles Drummer, Anthony Virtue, Tracy Gao, Susu Wu, Miguel Hernandez, Lexy Singh, Hong Wang, Xiao-Feng Yang

**Affiliations:** Center for Metabolic Disease Research, Cardiovascular Research Center, Lewis Katz School of Medicine at Temple University, Philadelphia, PA, United States

**Keywords:** microRNAs, atherosclerosis, diabetes, fatty liver disease, obesity, resistin, miR-155, miR-221

## Abstract

We reported that microRNA-155 (miR-155) deficiency in ApoE-/- mice yields a novel metabolically healthy obese (MHO) model, which exhibits improved atherosclerosis but results in obesity, non-alcoholic fatty liver disease (NAFLD) without insulin resistance. Using experimental data mining approaches combined with experiments, we found that, among 109 miRNAs, miR-155, and miR-221 are significantly modulated in all four hyperlipidemia-related diseases (HRDs), namely atherosclerosis, NAFLD, obesity and type II diabetes (T2DM). MiR-155 is significantly upregulated in atherosclerosis and decreased in other HRDs. MiR-221 is increased in three HRDs but reduced in obesity. These findings led to our new classification of types I and II MHOs, which are regulated by miR-221 and miR-155, respectively. Western blots showed that the proinflammatory adipokine, resistin, is significantly increased in white adipose tissues (WAT) of the MHO mice, revealing our newly proposed, miR-155-suppressed “secondary wave inflammatory state (SWIS),” characteristic of MHO transition to classical obesity (CO). Taken together, we are first to show that MHO may have heterogeneity in comorbidities, and is therefore classified into type I, and type II MHOs; and that increased expression of resistin in miR-155-/- white adipose tissues may be a driver for SWIS in MHO transition to CO. Our findings provide novel insights into the pathogenesis of MHO, MHO transition to CO, hyperlipidemic pathways related to cancer, and new therapeutic targets.

## Introduction

**FIGURE 1:**
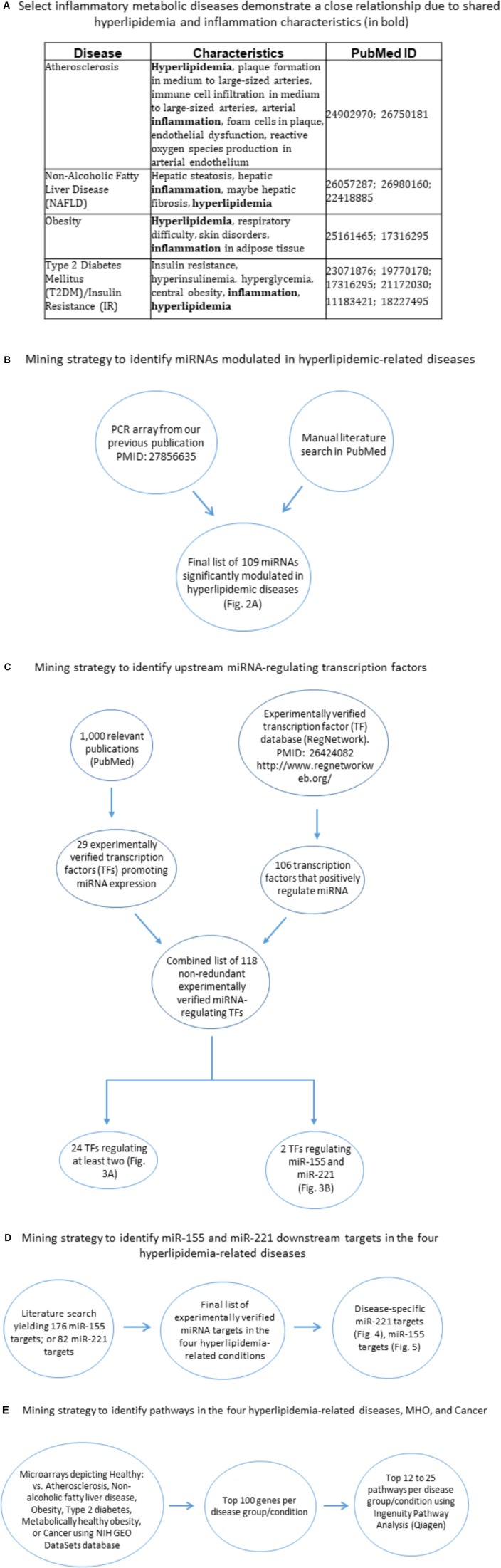
Flow chart of database mining strategy. **(A)** Select inflammatory metabolic diseases demonstrate a close relationship due to shared hyperlipidemia and inflammation characteristics. **(B)** Two methods of data mining were employed: PCR array data from our lab (PMID: 27856635) and literature search. **(C)** Two methods of data mining were employed. The first was analyzing 1,000 papers using PubMed, narrowing down to 66 relevant publications, yielding 29 experimentally verified TFs. The second method involved the use of the RegNetwork database, yielding 106 experimentally verified transcription factors. In all, a total of 135 overlapping transcription factors were obtained. This combined list was then assessed using two approaches: (1) In each single disease and co-morbid diseases group, TFs that regulated at least two miRNAs were chosen as significant and then overlapped to define unique and shared TFs; (2) identified transcription factors that regulate miR-155 and miR-221. **(D)** Literature search for miR-155 targets and miR-221 targets, which were then assessed whether they played roles in any of the four hyperlipidemia-related diseases. **(E)** Using NIH GEO DataSets database, microarray comparing healthy vs. disease human subjects; metabolic unhealthy vs. metabolically healthy obese patients were obtained. The top 100 significantly upregulated genes per condition were obtained and then the top 12–25 pathways per condition were obtained. Finally, these pathways were overlapped to identify shared pathways (**Figure 8**). TF, transcription factor.

Hyperlipidemia, or high low-density lipoprotein cholesterol, is a condition affecting 78 million Americans (High, 2017) and is a well-documented risk factor for a number of co-morbidities. These include atherosclerosis, obesity, non-alcoholic fatty liver disease (NAFLD), and type II diabetes mellitus (T2DM) (**Figure 1A**). Currently, the molecular mechanisms involved in the manifestation and progression of hyperlipidemia, particularly with respect to these co-morbidities, are not completely understood. MicroRNAs (miRNAs) are short (19–25 nucleotides), non-coding regulatory RNAs that regulate gene expression by mRNA translation inhibition or degradation (Virtue et al., 2012). In recent years, miRNAs have been firmly established as important regulators in a host of diseases, including cardiovascular disease, obesity, type II diabetes and NAFLD. Still, much remains to be understood regarding their overlap and function in hyperlipidemia-related pathologies. Over the years, multiple publications have shown the key roles that miRNAs play in these diseases. For example, we reported that a group of anti-atherogenic miRNAs may downregulate pro-atherogenic genes (Virtue et al., 2011). Soh et al. (2013) published their findings that miR-30c reduced atherosclerosis and hyperlipidemia in Western diet-fed mice. Hanin et al. (2017) found that transgenic miR-132-overexpressing mice resulted in fatty liver and hyperlipidemia. Vinnikov et al. (2014) showed that miR-103 secreted from the hypothalamus plays a role in preventing food-induced obesity. Latreille et al. (2014) reported that overexpression of miR-7a in pancreatic beta cells led to type II diabetes development. Additionally, in our recent paper (PMID: 27856635), we demonstrate that miRNA-155 is significantly increased in atherosclerosis but reduced in high-fat diet-induced obesity. We further presented the novel findings that global miR-155 deficiency in an atherosclerotic ApoE-/- mouse background reduced atherosclerosis but resulted in obesity, NAFLD, and hyperinsulinemia without insulin resistance. These findings demonstrate a suitable model for study of metabolically healthy obesity (MHO), a phenomenon that is present in up to 40% of the obese population (Hinnouho et al., 2013; Phillips and Perry, 2013). Metabolically healthy obesity (MHO), as the name suggests, is a condition whereby obese patients exhibit a healthy metabolic profile in that they are afflicted with none of the following commonly associated co-morbidities: elevated blood pressure, high fasting glucose levels, elevated triglycerides, and reduced high density lipoprotein levels (Ortega et al., 2016). Our prior experimental findings implied that a MHO regulator must have the following features: (1) a potent role of a single molecule (e.g., miRNA) to differentially affect multiple hyperlipidemia-related diseases (HRDs) such that this molecule (e.g., miRNAs) achieves master gene status; and (2) differential expressions and functions of downstream targets of the molecule (e.g., miRNA) in different cell types to carry out the following three functions: (a) promotion of atherosclerosis and vascular inflammation, but (b) suppression of white adipose tissue obesity; and (c) inhibition of MHO transition to classical obese-required adipokine(s). In our study, we show that the miRNAs such as miR-155 meet these criteria. However, the important issue that remains is the identification of the miRNAs that regulate common pathways shared between HRDs as well as pathways specific to each HRD.

Furthermore, we sought to expand our understanding of the various miRNAs in these HRDs, specifically in regards to their expression, regulation, and function. We hypothesized that miRNAs function as a potent master regulator that directs disease pathogenesis where only a few are shared between two or more hyperlipidemia-related conditions [e.g., atherosclerosis, obesity, type II diabetes mellitus/insulin resistance (T2DM/IR) and NAFLD]. Using microarray and other experimentally verified-based data mining approaches, we have found that, among 109 miRNAs, miR-155, and miR-221 are significantly modulated in all four HRDs. More specifically, we found that miR-155 is significantly upregulated in atherosclerosis and decreased in NAFLD, type II diabetes/insulin resistance (T2DM/IR), and obesity. We also found that miR-221 is increased in atherosclerosis, NAFLD and T2DM/IR but reduced in obesity. These findings led to our newly classified type I and II MHO, which we propose to be regulated by miR-221 and miR-155, respectively. Also known as “resistance to insulin,” resistin is a cysteine-rich hormone that is produced mainly by murine adipocytes. Resistin is positively associated with obesity, T2DM, atherosclerosis and NAFLD (Steppan et al., 2001; Rajala et al., 2004; Jamaluddin et al., 2012; Badoer et al., 2015). We previously showed that resistin is significantly increased in the plasma of miR155-/-/ApoE-/- MHO mouse model compared with ApoE-/- mice. To corroborate our newly proposed Type II MHO model, we show that the proinflammatory adipokine, resistin, is significantly increased in adipose tissue of our MHO mice following high-fat diet (HFD) feeding, revealing a miR-155-suppressed adipose-tissue generated inflammatory state, characteristic of MHO transition to classical obesity (Jung et al., 2017). Our results also show a significant role for three transcription factors (TFs), early growth response 1 (EGR1), NFκB and Signal transducers and activators of transcription 3 (STAT3), as upstream regulators for these and other miRNAs. Our data mining results identified mRNA targets that could contribute to the disease manifestations. Additionally, we identified groups of miRNAs that are unique to one HRD type as well as miRNAs that were modulated in comorbidities (i.e., two or more HRDs), which may be useful as future disease biomarkers. Taken together, we are first to show that NFκB- and STAT3-upregulated miR-221 and miR-155 potentially serve as master regulators for type I MHO and type II MHO, respectively, in addition to being regulators for four co-morbid HRDs. Additionally, we show that miR-155 expression in aorta, liver, and white adipose tissues; and that its downstream targets confer upon deficiency of miR-155 master regulator status in MHO.

## Materials and Methods

### miRNAs, miRNA-Regulating Transcription Factors, miRNA-Targeted mRNAs Studied

Compilation of miRNAs was done by manual literature search of the PubMed-NCBI database and by PCR array conducted in our lab. The search yielded a final list of 109 miRNAs that are significantly modulated in the four diseases. We identified miRNAs based on their expression levels in each disease condition. Compilation of microRNA-regulating transcription factors (TFs) was based on: (1) manual literature search through PubMed-NCBI database; and (2) the experiment-based RegNetwork: Regulatory Network Repository database (PMID: 26424082^1^). In the first method, we analyzed 1,000 peer-reviewed published experimental papers using PubMed, then narrowing down to 66 relevant publications, which yielded 29 experimentally verified TFs (i.e., TF-miRNA promoter interaction via luciferase assay). In the second method, using the RegNetwork database, we found 106 experimentally verified transcription factors. In all, a total of 135 overlapping transcription factors were obtained, which were then finalized to 118 TFs after removing duplicates. This combined list was then assessed using two approaches: (1) In each single and co-morbid disease group, transcription factors that regulated at least two miRNAs were chosen as significant; (2) we identified transcription factors that regulate miR-155 and miR-221. Compilation of miR-221/miR-155 mRNA targets was done by manual literature search of experimentally verified targets and miRTarBase (Chou et al., 2018), yielding 176 (miR-155) and 82 (miR-221) mRNA targets. These targets were then identified in the four HRDs by manual literature search, yielding 11 relevant mRNA targets for miR-221 and 20 for miR-155 (**Figures 1B–D**).

### Pathway Analyses

Pathway analyses were produced using Ingenuity Pathway Analysis (IPA, Qiagen): miRNAs having the same expression direction were analyzed in each category (e.g., all miRNAs that are significantly increased in atherosclerosis had their own category; all miRNAs that are significantly decreased in atherosclerosis had their own category). The designation up or down was then given, which reflected the expression direction of the miRNAs (**Supplementary Figure 1**). Compilation of pathways in the four HRDs, cancer and metabolically healthy disease (MHO) was done using NIH Gene Expression Omnibus (GEO) DataSets-NCBI database^2^ to compare microarrays for healthy vs. disease human subjects; metabolically unhealthy vs. metabolically healthy obese (MHO) patients. The top 100 significantly upregulated genes per condition were obtained; and then the top 12–25 pathways [depending on statistical significance (*p* < 0.05)] per condition were obtained. Finally, these pathways were overlapped between diseases/conditions to identify shared pathways using IPA and Venn diagram analysis (**Figures 1E**, **7**).

### Experiment Study

All animal experiments were performed in accordance with the Institutional Animal Care and Use Committee (IACUC) Guidelines and Authorization for the use of Laboratory Animals and were approved by the IACUC of Lewis Katz School of Medicine at Temple University, which conform to the National Institutes of Health Guidelines for the Care and Use of Laboratory Animals. Apolipoprotein E (ApoE) knockout mice, miR-155 knockout mice, and wild type (WT) mice were of a C57BL/6 background, and purchased from the Jackson Laboratory (Bar Harbor, ME, United States). Mice were housed in the LKSOM Animal Facility under controlled conditions, where they were given *ad libitum* access to standard chow diet and water, and put on a 12 h light-dark cycle. MiR-155-/-/ApoE-/- (DKO) mice were generated as previously reported (Virtue et al., 2017). At 8 weeks old, mice either remained on normal chow diet (5% fat, Labdiet 5001, St. Louis, MO, United States) or switched to high fat diet [0.2% (w/w) cholesterol and 21.2% (w/w) fat (TD. 88137, Harlan Teklad, WI, United States)] for 12 weeks.

Quantitation of protein level by Western blot was performed as previously reported (Virtue et al., 2017) on epididymal white adipose tissue from 20-week-old mice following 12 weeks of HFD. Antibodies to resistin (1:500, cat: ab119501) and beta-actin (1:2000, cat: ab8227) were purchased from Abcam; anti-rabbit IgG secondary antibody (1:2000, cat: 7074) were purchased from Cell Signaling.

All statistical analyses were performed with the GraphPad Prism 5 software. Quantitation data analysis was performed using One-Way ANOVA.

## Results

### miRNA Expression Profile Shows That Only a Few Shared miRNAs Are Potentially Crucial Regulators of HRDs

Metabolic syndrome is a condition that refers to collective risk factors that increase an individual’s likelihood of developing cardiovascular disease; and is an increasingly common diagnosis in the United States (Moore et al., 2017). Conditions that constitute metabolic syndrome include obesity and insulin resistance, while those resulting from metabolic syndrome include NAFLD, T2DM (Srikanthan et al., 2016) and atherosclerosis (González-Navarro et al., 2008). Largely having unique distinct features that define each disease, inflammation and hyperlipidemia stand out as shared characteristics of the four diseases (**Figure 1A**). Moreover, these four diseases continue to be prevalent within American society. Therefore, synthesizing a more comprehensive understanding of the molecules and mechanisms involved in these disease processes is requisite.

**FIGURE 2:**
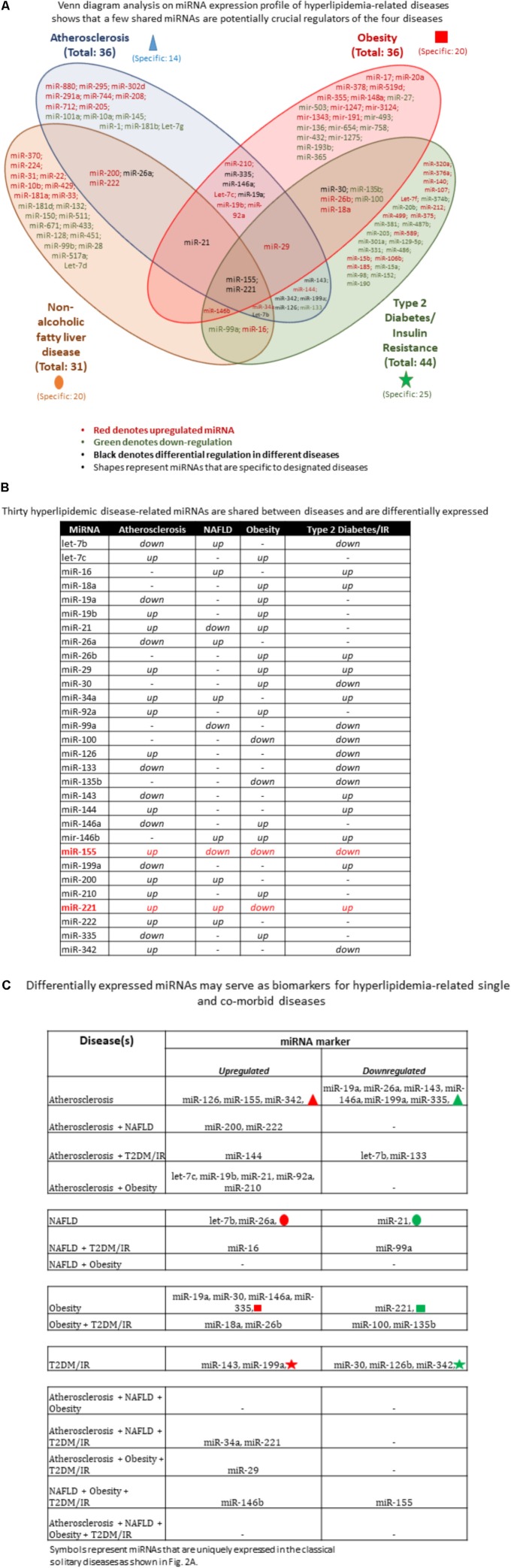
Hyperlipidemic diseases-shared miRNAs are fewer than disease-specific miRNAs. **(A)** miRNA expression profile of hyperlipidemia-related diseases shows that a few shared miRNAs are potentially crucial regulators of the four diseases. The Venn diagram analysis showed that the majority of miRNAs are uniquely expressed in certain pathologies while a few are shared across diseases. These shared miRNAs likely play an important regulatory role in disease development. **(B)** Hyperlipidemic disease-related miRNAs are differentially expressed. MiRNAs that are shared among diseases show different expression patterns, suggesting that upstream regulators are differentially expressed under varying disease conditions. Moreover, with more diseases, there are fewer miRNAs shared. **(C)** Differentially expressed miRNAs can be used as biomarkers for hyperlipidemia-related single and comorbid diseases. Using the shared miRNAs from **(A)**, we identified novel markers for comorbid diseases. The majority of the miRNA markers are upregulated miRNAs compared with downregulated miRNAs. Symbols represent miRNAs that are uniquely expressed in the classical solitary diseases as shown in **(A)** NAFLD, non-alcoholic fatty liver disease; IR, insulin resistance; T2DM, type 2 diabetes mellitus.

MicroRNAs play an indispensable role in the development of the hyperlipidemia-related pathologies atherosclerosis, NAFLD, obesity and T2DM. Thus, we wanted to determine the miRNAs that were shared among diseases and were unique to a disease. Since our previous publication highlighted that deficiency of a single master miRNA can produce drastic and opposing phenotypes, we hypothesized that only a few master miRNAs would be shared, leaving the vast majority of miRNAs unique to a single disease. We employed a literature search approach to determine experimentally verified miRNAs as well as used data generated from our microarray (**Figure 1B**). Using Venn diagram analysis to easily determine disease-unique miRNAs vs. master miRNAs shared among diseases, we found that of 109 miRNAs that were shown to be significantly upregulated or downregulated in any of the four diseases, the vast majority of miRNAs were uniquely modulated within each disease type while only a few master miRNAs were shared (**Figure 2A**). In terms of percentage, out of a total of 36 atherosclerosis-relevant miRNAs, we found that 39% were atherosclerosis-specific miRNAs, while 61% of all atherosclerosis-relevant miRNAs were shared (i.e., these miRNAs are relevant in atherosclerosis and one or more other diseases). Out of 31 NAFLD-relevant miRNAs, NAFLD-specific miRNAs accounted for 64.5% while shared miRNAs were 35.5%. Out of 36 obesity-relevant miRNAs, unique miRNAs were 52.7% while shared were 47.3%. Out of 44 T2DM/insulin resistance (T2DM/IR)-relevant miRNAs, 56.8% were unique miRNAs while 43.2% were shared miRNAs.

Some shared miRNAs were consistently expressed in the same direction while others were differentially expressed between diseases (shown in black color, **Figure 2A**). For example, the differentially expressed let-7b was significantly reduced in atherosclerosis and T2DM/IR but increased in NAFLD. MiR-19a, miR-146a, and miR-335 were reduced in atherosclerosis but increased in obesity. MiR-21 was increased in both atherosclerosis and obesity but decreased in NAFLD. MiR-26a was reduced in atherosclerosis and increased in NAFLD. MiR-30 was upregulated in obesity but downregulated in T2DM/IR, whereas both miR-126 and miR-342 were increased in atherosclerosis and reduced in T2DM/IR. On the contrary, miR-143 and miR-199a were decreased in atherosclerosis but increased in T2DM/IR. Finally, both miR-155 and miR-221 were increased in atherosclerosis but decreased in obesity, while differentially expressed in NAFLD and T2DM/IR (**Figure 2B**).

Moreover, we identified miRNAs that are shared by at least three diseases. MiR-21 was upregulated in atherosclerosis and obesity but downregulated in NAFLD; miR-29 was increased in atherosclerosis, obesity and T2DM/IR; miR-146b was significantly expressed in NAFLD, obesity and T2DM/IR; miR-34a was increased in atherosclerosis, NAFLD, and T2DM/IR, while let-7b was reduced in atherosclerosis and T2DM/IR but increased in NAFLD (**Figure 2B**). Most interestingly, miR-155 and miR-221 were the only master miRNAs that were found to be significantly modulated in all four pathologies, with miR-155 being significantly increased in atherosclerosis and reduced in the remaining three diseases while miR-221 was upregulated in atherosclerosis, NAFLD and T2DM/IR but lower in the obese phenotype.

### Differentially Expressed miRNAs May Serve as Biomarkers for Hyperlipidemia-Related Single and Shared Diseases

Based on miRNA analyses (**Figures 2A,B**), we were able to classify miRNAs as two types of biomarkers for (i) single disease condition; and (ii) for two or more co-existing, or shared, diseases. These miRNA markers were also categorized as upregulated and downregulated. For patients diagnosed with only atherosclerosis, associated upregulated miRNAs include miR-126, miR-155, and miR-342, and miRNAs represented by the red triangle symbol. Similarly, associated downregulated miRNAs include, for example, miR-19a, miR-335, and other miRNAs designated by the green triangle symbol. For patients diagnosed with only NAFLD, associated upregulated miRNAs included let-7b and miR-26a, along with other miRNAs represented by the red oval symbol, while miR-21 and miRNAs represented by green oval symbol were reduced. For obese patients, associated miRNAs included increased expression of miR-19a, miR-30, miR-146a, miR-335, and the miRNAs represented by the red square symbol; while miR-221 along with miRNAs depicted by green square symbol were decreased. Lastly, for T2DM/IR patients, associated miRNAs included increased miR-143, miR-199a, and the miRNAs represented by the red star symbol as well as reduced miR-30, miR-126b, miR-342, and miRNAs depicted by the green star symbol. Please note that symbols represent miRNAs that were uniquely expressed in the classical solitary diseases as shown in **Figure 2A**. For patients having co-morbid conditions, the miRNA markers were understandably fewer and therefore more specific. In patients having both atherosclerotic and NAFLD conditions, possible miRNA markers were miR-200 and miR-222. An atherosclerotic and T2DM/IR condition was marked by increased miR-144 but reduced let-7b and miR-133. An atherosclerotic and obese condition was associated with increased let-7c, miR-19b, miR-21, miR-92a, and miR-210. Patients diagnosed with a NAFLD-T2DM/IR co-morbid condition, we propose, would exhibit increased miR-16 and decreased miR-99a. An obesity and T2DM/IR condition was marked by increased miR-18a, miR-26b but reduced miR-100 and miR-135b. MiR-221 and miR-34 were upregulated markers in the atherosclerosis, NAFLD, T2DM/IR condition, whereas miR-29 was increased in atherosclerosis, obesity and T2DM/IR condition. Lastly, NAFLD, obesity, and T2DM/IR exhibit increased miR-146b but reduced miR-155 (**Figure 2C**). Interestingly, there were no shared miRNAs between only NAFLD and obesity; atherosclerosis, NAFLD and obesity; or atherosclerosis, NAFLD, obesity, and T2DM/IR that were significantly expressed in the same direction (i.e., no miRNAs were all increased or all decreased in these co-morbidities).

As mentioned earlier, miR-155 and miR-221 were the only miRNAs modulated in all four HRDs; and miR-155 deletion in our ApoE-/- mice led to a new MHO model, whereby obesity, NAFLD, and hyperinsulinemia without insulin resistance were present along with reduced atherosclerosis. We observed in **Figure 2B** that deficiency of miR-221 may result in MHO as well, since it is significantly upregulated in atherosclerosis, NAFLD, and T2DM/IR but reduced in obesity.

### High-Fat Diet Feed Significantly Upregulates the Protein Levels of Proinflammatory Adipokine, Resistin, in Type II MHO White Adipose Tissue

Resistin is a pro-inflammatory adipokine secreted by adipocytes in mice, that is associated with obesity and type 2 diabetes (Steppan et al., 2001; Rajala et al., 2004; Jamaluddin et al., 2012). Previously, we reported that resistin is significantly increased in the plasma of miR155-/-/ApoE-/- MHO mouse model compared with ApoE-/- mice (Virtue et al., 2017). In order to confirm our newly proposed Type II MHO model, i.e., the designation of MHO being converted to classically unhealthy obesity, we probed for pro-inflammatory resistin in adipose tissue of MHO model. We found that 12 weeks of HFD-fed MHO mice had significantly higher pro-inflammatory resistin levels in white adipose tissue compared with that of atherosclerotic ApoE-/- mice and that of diet-induced classically obese WT mice (**Supplementary Figure 2**). These results demonstrated that (1) increased plasma resistin is derived from increased expression of resistin in white adipose tissues; (2) resistin expression is suppressed by miR-155, thus resistin expression is increased in miR-155 DKO white adipose tissues; and (3) since resistin is a proinflammatory adipokine, high-fat diet induction of resistin upregulation in white adipose tissues makes resistin a potential significant mediator to drive “a second wave of inflammation” in the miR-155-/- MHO status, which is required to MHO transition to classical obesity.

### The Majority of Transcription Factors Are Specific to Single Disease Conditions (i.e., Atherosclerosis, NAFLD, Obesity or T2DM) While Only a Few Transcription Factors Are Shared Among Co-existing Disease Conditions (i.e., at Least Two HRDs)

**FIGURE 3:**
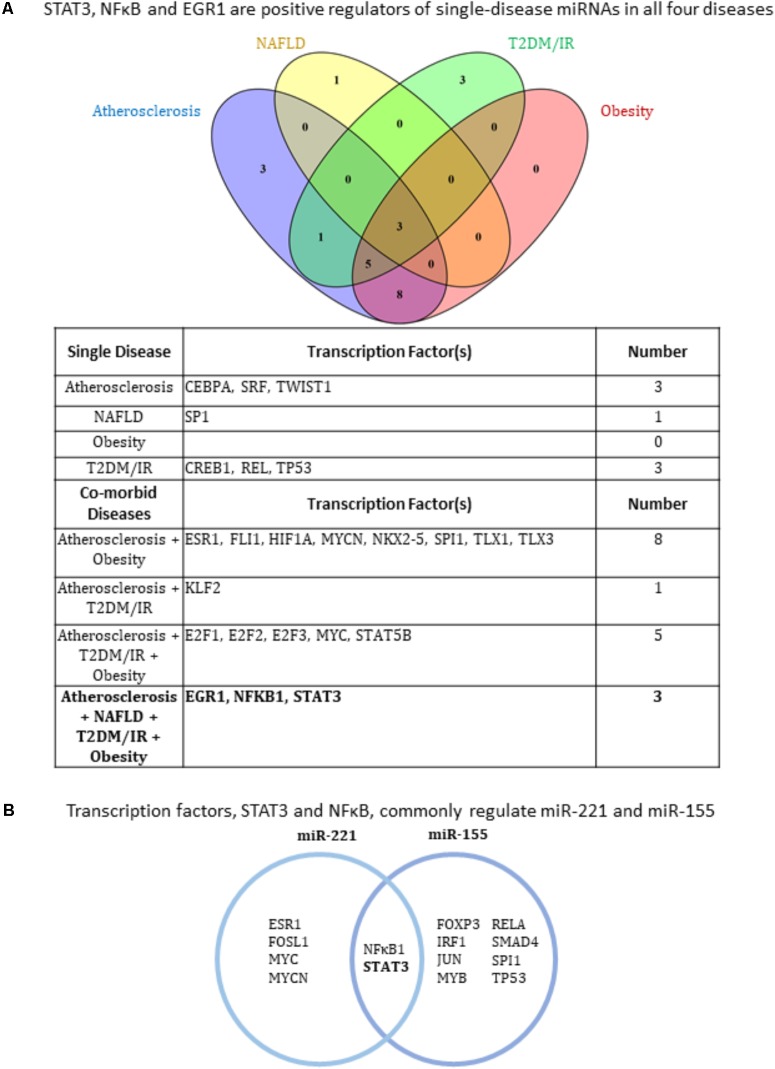
Three transcription factors EGR1, NFκB and STAT3 regulate miRNAs related to all four hyperlipidemia-related diseases. **(A)** Based on miRNAs found in **Figure 2**, experimentally verified transcription factors increasing their expression were collected and those regulating more than one miRNA gene from each single and co-morbid disease cluster are shown. The diagram shows EGR1, NFκB, and STAT3 regulate miRNAs in all four disease clusters. NAFLD, non-alcoholic fatty liver disease. T2DM/IR, type 2 diabetes mellitus/insulin resistance. **(B)** STAT3 and NFκB are key miR-155 and miR-221-regulating transcription factors. Manual publication search and database mining approach show that pro-inflammatory transcription factors, STAT3 and NFκB, positively regulate the expression of miR-155 and miR-221.

Transcription factors (TFs) play a critical role in regulating the expression of protein-coding genes, among them of course other TFs. These all-important proteins also regulate the expression of non-coding RNAs such as miRNAs in the same way as they do protein-coding genes, i.e., by binding to their promoters to either inhibit or promote transcription. We next researched the TFs that have been experimentally verified to bind the promoter or enhancer regions of each miRNA gene in order to elicit an increase in miRNA expression. In other words, we included only TFs shown to promote the miRNAs’ expression. In terms of methodology, we conducted two approaches to identify TFs. In the first approach, for each disease, we included TFs that regulated at least two miRNAs within each disease type as well as within co-existing disease combination groups. The second approach we employed was to identify TFs that commonly regulated the only miRNAs that are modulated in all four disease types: miR-155 and miR-221. Using these approaches, we found that three transcription factors, EGR1, NFκB (Huang et al., 2013) and STAT3, were regulators of miRNAs in all four diseases (**Figures 3A,B**). With either approach, NFκB and STAT3 were positive regulators of miRNAs in all four diseases (**Figures 3A,B**), while EGR1 was also found to be a positively regulator using the first approach (**Figure 3A**).

### Downstream mRNA Targets of Master miR-155 and miR-221 in Atherosclerosis, NAFLD, Obesity and T2DM/IR Are Largely Unique to Each miRNA; And Are Differentially Expressed in Each of the Four Diseases, Leading to Different Phenotypic Outcomes

**FIGURE 4:**
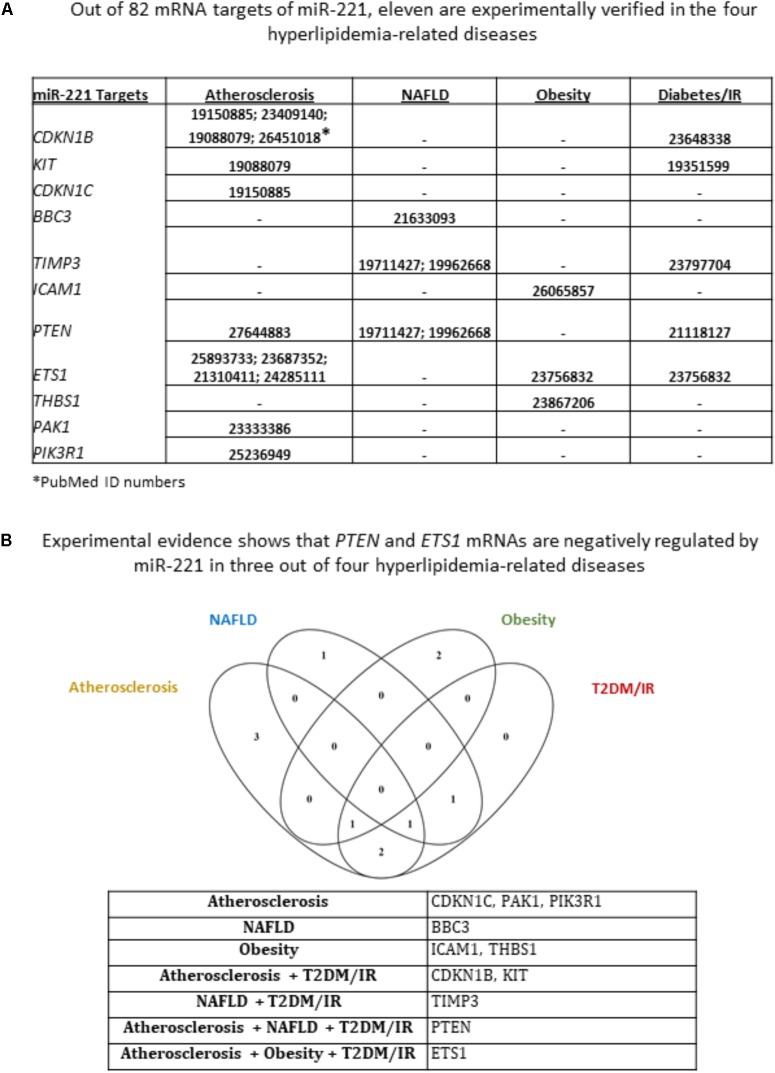
Of 11 miR-221 mRNA targets that have been experimentally verified in any of the four hyperlipidemia-related disease backgrounds, PTEN and ETS1 mRNAs are negatively regulated in three out of four hyperlipidemia-related diseases. **(A)** From an original list of 82 miR-221 mRNA targets, we identified via PubMed the targets that were tested in any of the four diseases. We found 11 mRNA targets met this criterion. **(B)** Moreover, few mRNA targets were shared between diseases; and Venn diagram shows that PTEN and ETS1 mRNAs are negatively regulated by miR-221 in three out of four hyperlipidemia-related diseases. NAFLD, non-alcoholic fatty liver disease; T2DM/IR, type 2 diabetes mellitus/insulin resistance.

**FIGURE 5:**
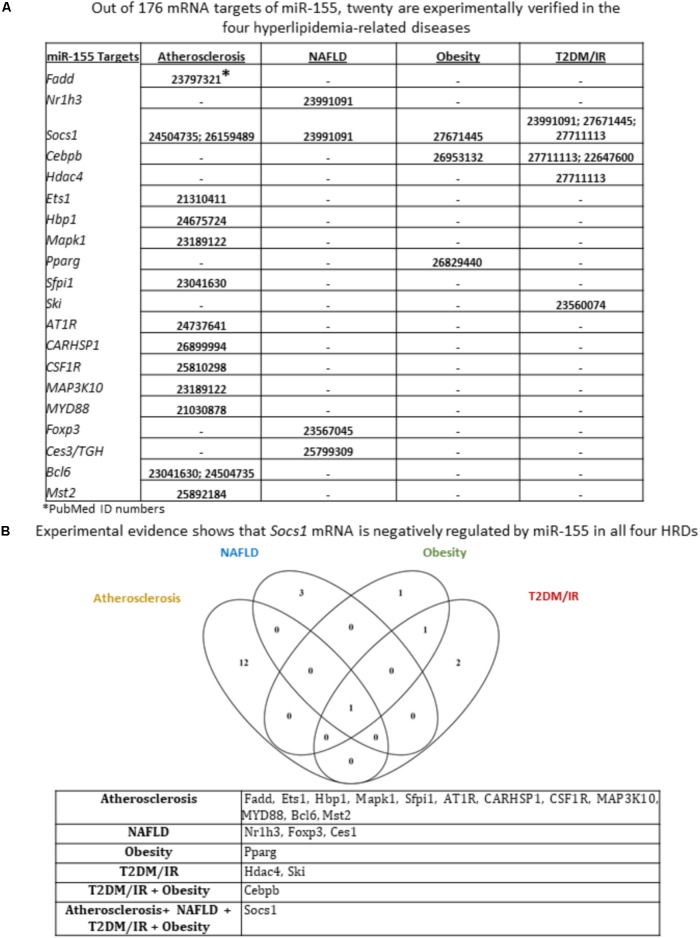
Of 20 miR-155 mRNA targets that have been experimentally verified in any of the four hyperlipidemia-related disease backgrounds, only Socs1 mRNA is shown to be negatively regulated by miR-155 in all four disease contexts. **(A)** From an original list of 176 miR-155 mRNA targets, we identified via PubMed the targets that were tested in any of the four diseases. We found 20 mRNA targets met this criterion. **(B)** Moreover, few mRNA targets were shared between diseases; and Venn diagram shows that Socs1 is negatively regulated by miR-155 in all four hyperlipidemia-related diseases. NAFLD, non-alcoholic fatty liver disease; T2DM/IR, type 2 diabetes mellitus/insulin resistance; HRDs, hyperlipidemia-related diseases

We next sought to identify miR-155 and miR-221 mRNA targets that play a role in the development of atherosclerosis, NAFLD, obesity and T2DM/IR. Of note, due to a lack of microarray data associated with MHO, we could not determine the mRNA targets for those two miRNAs in this condition. We hypothesized that varying phenotypes resulting from miR-155 deletion in our atherosclerotic mice resulted from varying expression of downstream targets (**Supplementary Figure 3**). From 82 known mRNA targets of miR-221, we found that eleven were experimentally verified in one or more of the four HRDs. These were cyclin-dependent kinase inhibitor 1B (CDKN1B); CDKN1C; KIT proto-oncogene receptor tyrosine kinase (KIT); BCL2 binding component 3 (BBC3); TIMP metallopeptidase inhibitor 3 (TIMP3); intercellular adhesion molecule 1 (ICAM1); phosphatase and tensin homolog (PTEN); ETS proto-oncogene 1, transcription factor (ETS1); thrombospondin 1 (THBS1); P21 (RAC1) activated kinase 1 (PAK1); and phosphoinositide-3-kinase regulatory subunit 1 (PIK3R1) (**Figure 4A**). While none of the targets were experimentally verified in all four diseases, we found that PTEN was targeted in atherosclerosis, NAFLD and T2DM/IR, while ETS1 was targeted in atherosclerosis, obesity and T2DM/IR (**Figure 4B**). From 176 mRNA targets of miR-155, 20 were experimentally verified in one or more of the four HRDs: Fas associated via death domain (Fadd); nuclear receptor subfamily 1 group H member 3 (Nr1h3); suppressor of cytokine signaling 1 (Socs1); CCAAT/enhancer binding protein beta (Cebpb); histone deacetylase 4 (Hdac4); Ets1; HMG-Box transcription factor 1 (Hbp1); mitogen-activated protein kinase 1 (Mapk1); peroxisome proliferator activated receptor gamma (Pparg); Spi-1 proto-oncogene (Sfpi1); SKI proto-oncogene (Ski); angiotensin II receptor type 1 (AT1R); calcium regulated heat stable protein 1 (CARHSP1); colony stimulating factor 1 receptor (CSF1R); mitogen-activated protein kinase kinase kinase 10 (MAP3K10); myeloid differentiation primary response 88 (MYD88); Forkhead box P3 (Foxp3); carboxylesterase 3 (Ces3/TGH); B-cell CLL/lymphoma 6 (Bcl6); and serine/threonine kinase 3 (Mst2) (**Figure 5A**). Interestingly, Socs1 was a common target in all four disease types (**Figure 5B**). Moreover, ETS1 is a shared mRNA target of both miR-155 and miR-221 (**Figures 4**, **5**). These results demonstrated that miR-155 and miR-221 are the master miRs, which have a few features: (1) shared expression patterns in a few diseases; (2) regulation of the expression of a list of master regulators such as transcription factors; and (3) establishment of special phenotypes due to the miR-deficiencies-induced upregulation of tissue-specific expression of certain mRNA targets. These new principles were very well demonstrated in our new MHO mouse model (Virtue et al., 2017). The MHO phenotypes in miR-155 deficiency results in decreased global inflammation derived from upregulation of anti-inflammatory miR155 target suppressor of cytokine signaling 1 (SOCS1) and in white adipose tissue hypertrophy derived from upregulation of miR-155 targets, adipogenic transcription factors C/EBPα, C/EBP-β, and PPAR-γ (Virtue et al., 2017).

### The Pathway Analyses Show That Disease-Unique and Disease-Shared Groups Have Modulated Pathways in Cancer, Metabolism, Immunology, Arthritis, Proliferation/Survival, and Development

**FIGURE 6:**
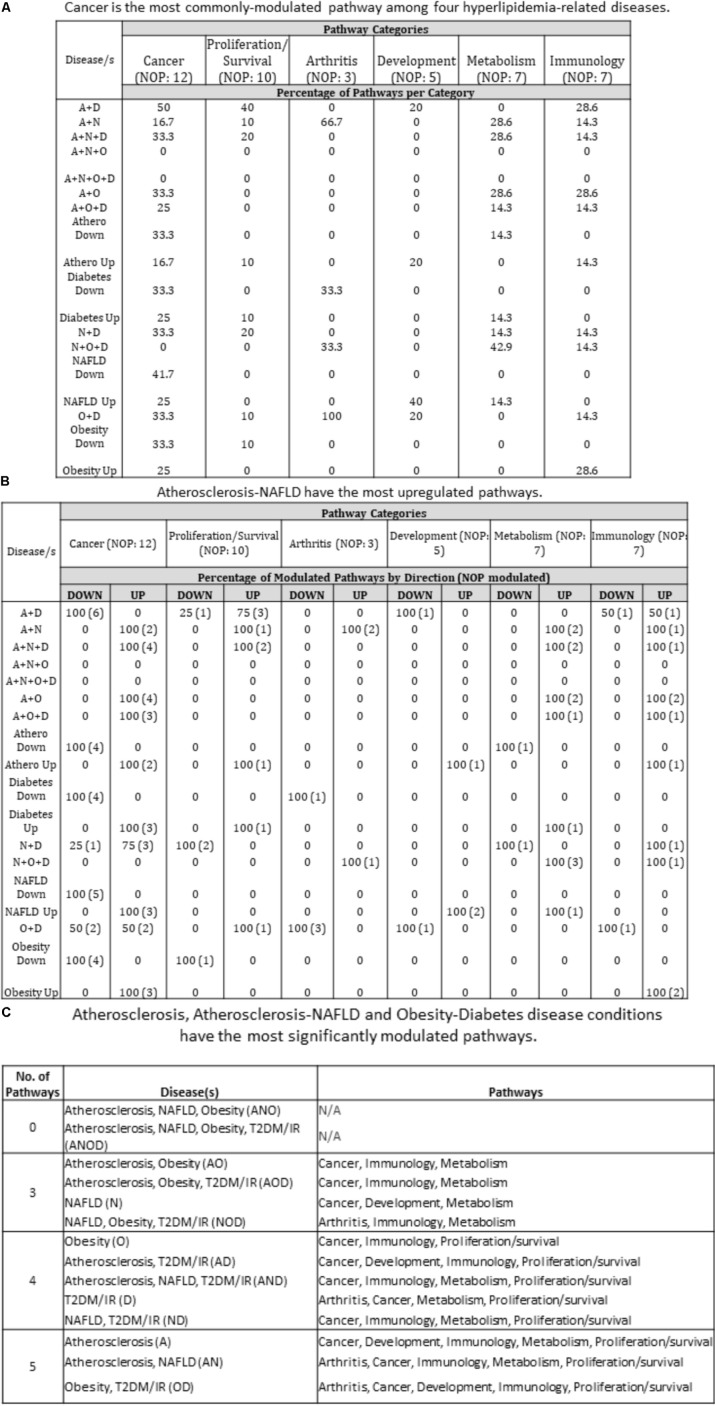
Pathway analyses of miRNA-regulating transcription factors show that unique and shared disease groups have six modulated pathways (cancer, metabolism, immunology, arthritis, proliferation/survival, development). **(A)** Cancer is the most commonly modulated pathway among diseases. Referring to **Supplementary Figure 2**, each number corresponds to the percentage of pathways that are modulated out of the total pathways per category. For example, in A + D co-morbid disease, 6 pathways are significantly modulated out of 12 total pathways in the Cancer pathways category. Therefore, 50% of cancer-related pathways are modulated. Most disease groups overlap with pathways related to Cancer. **(B)** Referring to **Supplementary Figure 2**, percentage of upregulated and downregulated pathways was determined from total number of modulated pathways per disease category. For example, under Cancer pathways, there were four modulated pathway subsets under N + D. Three of the four pathway subsets were upregulated and one was downregulated. Therefore, 75% of modulated pathway subsets was upregulated and 25% was downregulated under N + D. A + N disease group has the most upregulated pathways across the categories (five out of six categories). **(C)** Atherosclerosis, Atherosclerosis-NAFLD and Obesity-Diabetes disease conditions have the most significantly modulated pathways. U/D, up/down. U/D was not counted toward percentage; A, atherosclerosis; N, non-alcoholic fatty liver disease; O, obesity; D, diabetes; NOP, number of pathways.

We next decided to assess the pathways that each of these disease groupings are involved in. Employing Qiagen’s Ingenuity Pathway Analysis software, we found that each disease grouping [with the exception of ANO (atherosclerosis, NAFLD and obesity) and ANOD (atherosclerosis, NAFLD, obesity, and T2DM)] involved three or more of six pathway categories: Cancer, Proliferation/Survival, Arthritis, Development, Metabolism, and Immunology. As an example, in the NOD (NAFLD, obesity and T2DM/IR) condition, the pathways related to arthritis, immunology and metabolism were active. More specifically, molecules related to Role of Macrophages, Fibroblasts and Endothelial Cells in Rheumatoid Arthritis (Arthritis); Hepatic Cholestasis, Hepatic Fibrosis/Hepatic Stellate Cell Activation, PPAR Signaling (Metabolism); and INOS Signaling (Immunology) were increased, suggesting that chronic presence of NOD may possibly lead to the development of pathway-indicated pathologies (**Supplementary Figure 1** and **Figure 6C**). We next determined the percentage of pathway subsets that were significantly modulated among all pathway subsets within a category. For instance, referring to **Supplementary Figure 1A**, in AD (atherosclerosis and T2DM), six out of 12 total pathway subsets were modulated so that 50% of cancer-related pathways were modulated (**Figure 6A**). We therefore found that 15 out of 16 disease groupings (ANO and ANOD were excluded, since no miRNAs were expressed in the same direction in these co-morbidities) had modulated pathways in Cancer category. Next, we examined percentage of upregulated and downregulated pathways per disease group per pathway category. For example, under Cancer pathways, there were four modulated pathway subsets under ND. Three of the four pathway subsets were upregulated and the remaining one was downregulated. Therefore, 75% of modulated pathway subsets was upregulated and 25% was downregulated under ND. We found that AN had the most upregulated pathways across pathway categories (five out of six categories). On the other hand, AD and OD had the most downregulated pathways across pathway categories (four out of six categories) (**Figure 6B**). Interestingly, we identified atherosclerosis, the AN (atherosclerosis and NAFLD), and the OD (obesity and T2DM/IR) groupings were involved in pathways found in five out of six categories. All three had modulated pathways in Cancer, Immunology, and Proliferation/Survival pathway categories (**Figure 6C**). As stated earlier, miRNAs having the same expression direction were analyzed in each disease/combination category. However, we found no miRNAs that had the same expression direction in all four disease conditions.

### Top Pathways in HRDs, MHO, and Cancer Show Multiple Shared Signaling

**FIGURE 7:**
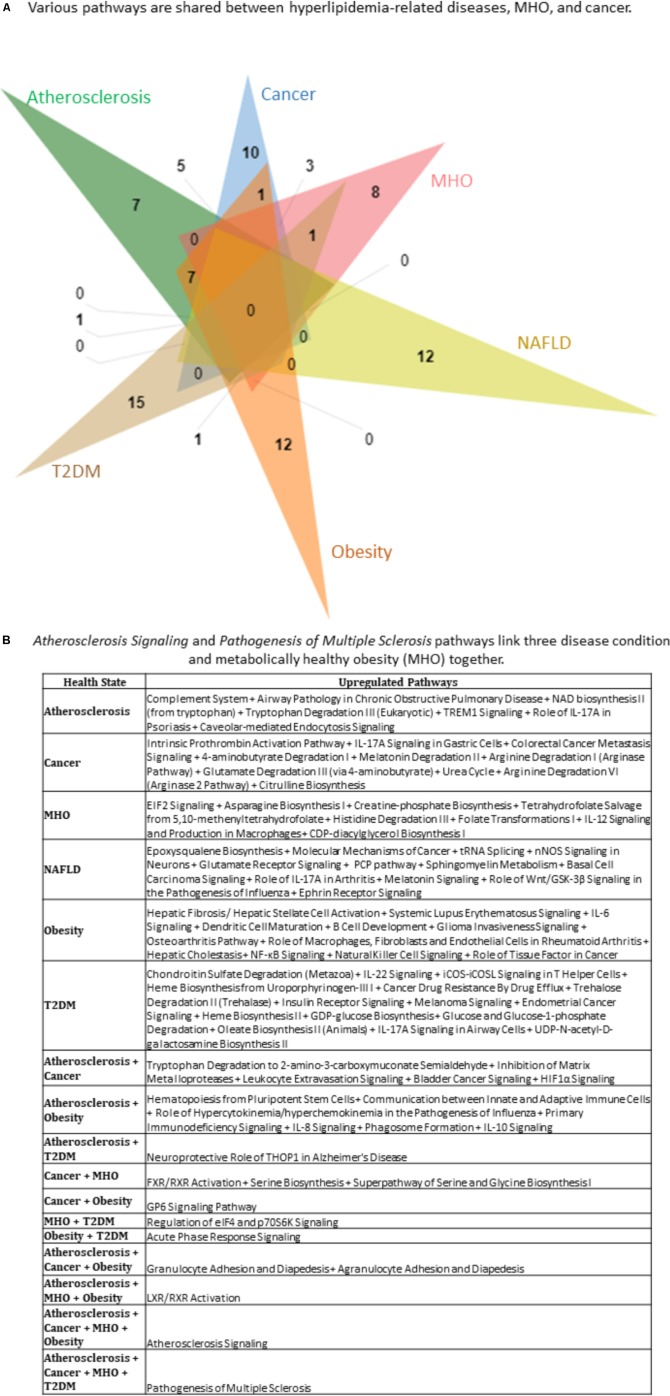
Top pathways in hyperlipidemia-related diseases, MHO, and cancer show multiple shared pathways. The top 25 (Atherosclerosis), 23 (Cancer), 15 (MHO), 12 (NAFLD), 25 (Obesity), 19 (T2DM) pathways were chosen. A 6-way Venn diagram was then constructed, which show that **(A)** various pathways are shared between hyperlipidemia-related diseases, MHO, and cancer. **(B)** Atherosclerosis Signaling and Pathogenesis of Multiple Sclerosis pathways link three disease conditions and MHO together. MHO, metabolically healthy obesity.

So far, we have studied HRDs as well as MHO. We have also found that the most modulated pathways are the cancer-related pathways (**Figure 6A**). Using the GEO DataSets database, we next compared microarrays (healthy vs. atherosclerotic human artery/plasma; healthy vs. NAF human liver; healthy vs. obese adipose tissue; healthy vs. diabetic human pancreas/blood/vessels/liver; healthy vs. malignant human tissue; and classically unhealthy obesity vs. MHO). The top 100 significantly upregulated genes per condition were obtained and then the top 12–25 pathways per condition were obtained. More specifically, the top 25 pathways (atherosclerosis); 12 pathways (NAFLD); 25 pathways (obesity); 19 pathways (T2DM); 15 pathways (MHO) and 23 pathways (cancer) were chosen based on significance (*p* < 0.05). These pathways were then overlapped to identify shared pathways (**Figures 7A,B**). While no pathways were shared among all six conditions, we found the following comorbidities had shared pathways: Atherosclerosis + Cancer; Atherosclerosis + Obesity; Atherosclerosis + T2DM; Cancer + MHO; Cancer + Obesity; MHO + T2DM; Obesity + T2DM; Atherosclerosis + Cancer + Obesity; Atherosclerosis + MHO + Obesity; Atherosclerosis + Cancer + MHO + Obesity; and Atherosclerosis + Cancer + MHO + T2DM. Furthermore, Atherosclerosis Signaling and Pathogenesis of Multiple Sclerosis pathways were shared among four out of six conditions (**Figure 7B**).

## Discussion

Since their discovery in 1993, miRNAs have proven to be important regulators of gene expression under both physiological and pathological conditions (Virtue et al., 2011, 2012), including atherosclerosis, obesity, T2DM/IR and NAFLD. Furthermore, over the years, it has become clear that hyperlipidemia undergirds the formation of and/or results from these four diseases. In addition, we now know that these diseases can mutually promote their development. For instance, obesity serves as a risk factor for the development of atherosclerosis, insulin resistance and increases deposition of fat in the liver, giving rise to NAFLD (Pi-Sunyer, 2009; Gaggini et al., 2013). Insulin resistance is a well-noted risk factor for type II diabetes; and the latter is a risk factor for atherosclerosis (Chait and Bornfeldt, 2009). With the increasing prevalence of these disease conditions, understanding their molecular underpinnings is imperative.

In our recently published manuscript (Virtue et al., 2017), we found that global miR-155 deletion in an atherosclerotic mouse background (ApoE knockout mice) improved atherosclerosis but resulted in obesity, NAFLD, and hyperinsulinemia without insulin resistance. These findings therefore support the establishment of our miR155-/-/ApoE-/- double knockout mice as a MHO model. This data (1) depicts the potent role of a single miRNA to differentially affect HRDs such that miRNAs achieve master gene status; and (2) implies a differential expression of downstream targets of miRNA in different cell types. On a whole, the focus of miRNA research has chiefly revolved around identifying the targets of miRNA, while upstream regulators of miRNAs have remained comparatively largely unstudied. Among the upstream regulators of miRNAs that have been studied, none have been studied in these HRDs to the best of our knowledge (**Supplementary Table 1**).

Based on our lab’s recently published findings, in this data mining study, firstly, we identified that significantly modulated miRNAs are largely specific to single diseases while only a few miRNAs are shared among disease clusters. While numerous miRNAs are expressed within and among various diseases, only miR-155 and miR-221 are significantly modulated in all four diseases; secondly, we found that only a few significantly modulated experimentally verified TFs regulate the miRNAs in shared diseases. Furthermore, the TFs regulating miRNAs in all four diseases include EGR1, NFκB, and STAT3; thirdly, these data suggest potential biomarkers for single and co-morbid diseases that may be used in predicting the patients at risk for developing one or more of these four diseases; fourthly, we compiled the downstream miRNA targets of miR155 and miR-221 that have been experimentally verified to promote or inhibit one or more of these diseases. These findings demonstrate that miRNAs, such as miR-155, are able to carry out their potent and differential functions due to presence and abundance of various mRNA targets expressed in various tissues. Of interest is the finding that SOCS1 is commonly targeted in all four diseases; fifthly, using IPA, we found that these diseases had active pathways in six categories: arthritis, cancer, development, immunology, metabolism, and proliferation/survival. We have also found that various HRDs share pathways with cancer and MHO, suggesting the possibility that these conditions are interrelated. Such a finding may imply that depending on the disease or comorbid diseases a patient has, he or she can be placed at risk for developing separate conditions related to the affected relevant pathway(s). Indeed, studies have reported an association between MHO and cancer. For instance, Sinn et al. (2017) showed that, compared to healthy, normal weight adults, MHO adults had increased risk of colorectal adenomas, a pre-malignant stage in colorectal cancer development. Similarly, compared with healthy, normal weight women, those with MHO had a higher risk of developing postmenopausal breast cancer (Moore et al., 2014). It is important to note that while MHO may still confer risk for cancer development, compared with metabolically unhealthy obese individuals, the risk is lower (Moore et al., 2014). As mentioned earlier, up to 40% of obese patients are metabolically healthy, thus these findings apply to a large portion of obese patients (Hinnouho et al., 2013; Phillips and Perry, 2013). As far as the remaining HRDs, it was reported that atherosclerotic women had a fivefold higher risk of developing lung cancer than non-atherosclerotic women (Dreyer et al., 2003). It is well-known that NAFLD is often a precursor stage of hepatocellular carcinoma but also colorectal cancer (Michelotti et al., 2013; Sanna et al., 2016). Additionally, obesity is known to have a positive correlation with over ten types of cancer (Obesity and Cancer National Cancer Institute, 2017). An increased risk of various cancer types is also observed in T2DM patients (Wang et al., 2015). These studies support our findings of an association between cancer and the various conditions covered in our study. Also, the shared pathways uncovered in our study show the possible mechanisms by which cancer and the various diseases are connected. Future experiments are needed to confirm that the uncovered pathways are indeed the mechanisms underlying the connection with cancer.

Not only do various reports provide a link between cancer development and the four HRDs, but these HRDs have also been associated with oxidative stress. More specifically, miRNAs in these four HRDs have been identified as protective against oxidative stress or as promoting oxidative stress. For instance, miR-210 (Ying et al., 2017) in atherosclerosis, miR-378 (Carrer et al., 2012) in obesity, miR-34a (Derdak et al., 2013) in NAFLD, miR-126 (Suresh Babu et al., 2016) in T2DM. These experimental findings demonstrate that miRNAs help to mediate protective effects against or damaging effects of oxidation species.

**FIGURE 8:**
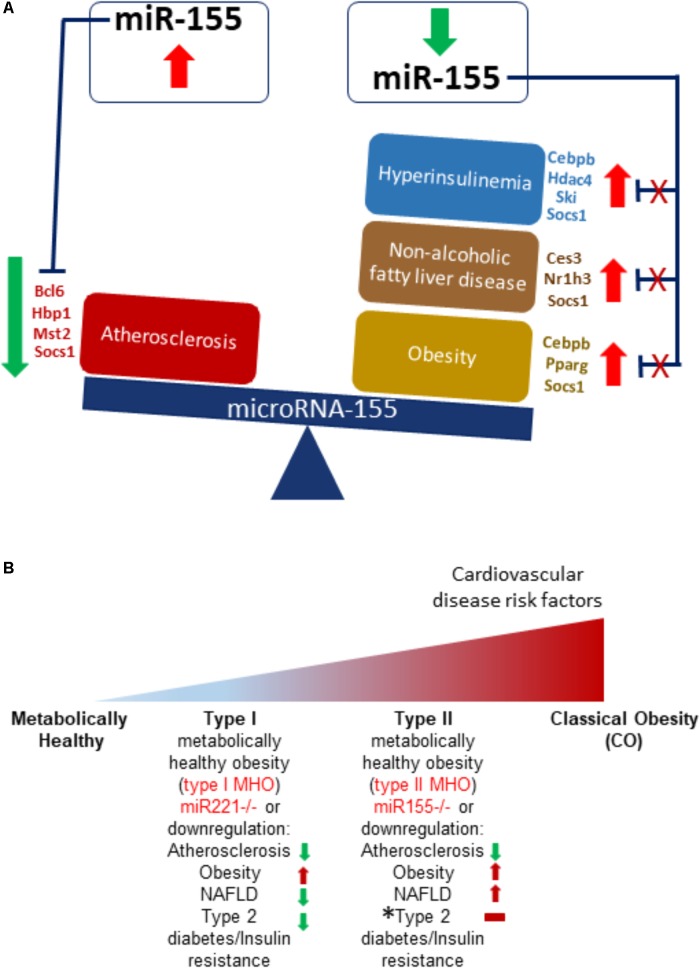
Transcription factors, such as STAT3, can promote miR155 transcriptional levels, which then target varied protein-encoding mRNA levels in tissues to create different and opposing phenotypes in different tissues. **(A)** Atherosclerotic apoE-deficient mice present with atherosclerosis in an otherwise normal phenotype in liver, adipose tissue and circulating insulin levels. This phenotype may be due to, for Instance, increased STAT3 levels that increase miR155 transcript levels that then target tissue-specific genes, creating paradoxical outcomes. MiR155 deficiency in atherosclerotic apoE-knockout mice display reduced atherosclerosis despite obesity, non-alcoholic fatty liver disease, and hyperinsulinemia. Reduced STAT3 and other miR-155-driving transcription factors yield decreased miR155 levels, yielding increased target mRNA levels and a change in the ApoE-/-/miR-155-/- double knockout mouse’s phenotypes. **(B)** MHO exists as subtypes. In the progression from healthy lean to the impermanent MHO to CO, we propose that miR-221 is first to be downregulated to create type I MHO, with features of obesity but no atherosclerosis, NAFLD, or T2DM/IR. Eventually, Type II MHO follows, which is due to miR-155 downregulation. This manifests as obesity with NAFLD and hyperinsulinemia, but no cardiovascular disease. Over time, MHO makes the transition to classically unhealthy obesity, which is characterized by cardiovascular disease, NAFLD, T2DM/IR, along with obesity. MHO, metabolically healthy obesity. ^∗^Horizontal red bar indicates that Type 2 diabetes/Insulin Resistance is not achieved but due to hyperinsulinemia in our miR155/apoE double knockout model, there is a trend in this direction.

Early growth response 1 is a transcription factor that in normal conditions has low or no expression but is associated with inflammation due to injury where it can promote wound healing or pathological fibrosis. Furthermore, this TF plays a role in cancer and schizophrenia development and is a member of the immediate and fast transcription group of genes known as immediate early genes (IEGs). Prior studies show increased expression of EGR1 is associated with atherosclerosis (Bhattacharyya et al., 2013) while EGR1 knockout mice exhibited resistance to obesity, insulin resistance and fatty liver (Zhang et al., 2013). Signal transducers and activators of transcription 3 plays a role in apoptosis, cellular proliferation, angiogenesis, cancer development, and inflammation (Mali, 2015). Phosphorylation of STATs is needed for their dimerization and subsequent transcription factor activity (Villarino et al., 2015). In atherosclerosis, STAT3 is activated in response to oxidized LDL, an initiator molecule of atherosclerosis (Mazière et al., 1999). Interestingly, suppressor of cytokine signaling 1 is a negative regulator of STAT3 (Wang et al., 2016). Moreover, T cell-mediated STAT3 promoted insulin resistance and obesity (Priceman et al., 2013) while leptin (satiety hormone) signaling relied on downstream STAT3 signaling (Buettner et al., 2006). Furthermore, STAT3 is active in NAFLD (Min et al., 2015). NFκB is a pleiotropic TF that regulates cell death, proliferation, the immune response, and plays a role in a host of diseases (Hayden and Ghosh, 2012), including promoting atherosclerosis (Pamukcu et al., 2011), being associated with obesity, T2DM (Baker et al., 2011) and, as some evidence suggests, being associated with NAFLD (Zeng et al., 2014). Previous studies show that SOCS1 is suppressed in atherosclerosis (Yang et al., 2015) but promotes insulin resistance (Ueki et al., 2004). Furthermore, SOCS1 is an NFκB negative regulator (Fujimoto and Naka, 2010). Taken together, our findings demonstrate that STAT3 and NFκB may be critical mediators of the hyperlipidemia-related pathologies of atherosclerosis, NAFLD, obesity and type II diabetes/insulin resistance via miR-155, which being upregulated/present in mice having an atherosclerosis background, worsens atherosclerosis but does not significantly affect the health of the liver, adipose tissue, or blood glucose levels. However, regarding patients having MHO (i.e., obese with healthy cardiovascular condition), we propose that in such a situation, STAT3 and/or NFκB is reduced, leading to reduction of miR-155. This produces a phenotype of obesity, NAFLD, hyperinsulinemia without insulin resistance but reduced atherosclerosis. Our compiled data demonstrate that this paradoxical phenotype is achieved due to presence of different targets being expressed in the different cell types as well as differing levels of these targets (**Figure 8A**). More specifically, mRNA targets such as Bcl6, Hbp1, Mst2, and Socs1, which are reduced in atherosclerosis have been shown to play a protective role against the disease’s development. Cebpb, Hdac4, Ski, Socs1 mRNAs, which are reduced in diabetes and/or insulin resistance, have also demonstrated protective roles in these conditions. On the other hand, reducing Ces3, Nr1h3, Socs1 levels in NAFLD has been shown to be protective. Similarly, decreased levels of Cebpb, Pparg, and Socs1 mitigated diet-induced obesity (**Figure 5A**).

Various studies led us to conclude that MHO is not a static condition but a temporary transient state that eventually results in classically unhealthy obesity (**Figure 8B**) (Hinnouho et al., 2013; Jung et al., 2017). Previously, we found that miR-155 deficiency led to reduced atherosclerosis (and therefore cardiovascular risk factors) but increased obesity, NAFLD, and hyperinsulinemia without insulin resistance. Based on our current findings, we identify miR-221 deficiency as another MHO model as **Figure 2B** shows (reduced atherosclerosis, NAFLD, and T2DM but increased obesity), suggesting that global deficiency of miR-221 may lead to obesity without any of the other inflammatory and hyperlipidemic diseases. Indeed, numerous studies lend support to the pro-atherogenic role of elevated miR-221 (Kothapalli et al., 2013; Zhang et al., 2014; Coskunpinar et al., 2016; Mandraffino et al., 2017; Talepoor et al., 2017). Moreover, deficiency of miR-155 or miR-221 leads to two varied manifestations of MHO, based on which we have proposed a new concept of MHO heterogeneity and classification of MHO; herein labeled as Type 1 MHO (miR221-/-) or Type 2 MHO (miR155-/-) (**Figure 8B**). In alignment with studies showing MHO as a temporary state of obesity, our data suggests that miR-221 is first to be downregulated to create type I MHO. This is then followed by miR-155 downregulation, leading to type II MHO. Finally, MHO makes the transition to classically unhealthy obesity. We have shown that resistin, a pro-inflammatory hormone secreted by murine adipocytes, was increased in the adipose tissue of our MHO model following 12 weeks of HFD feeding compared with atherosclerotic ApoE-/- mice and classical obesity diet-induced obesity WT mice. We previously showed that MHO mice had increased plasma resistin levels compared with ApoE-/- mice following HFD (Virtue et al., 2017). Previous reports show that pro-inflammatory resistin is significantly increased in serum but reduced in the white adipose tissue of diet-induced as well as genetic models of obesity (Rajala et al., 2004; Lefterova et al., 2009). Our MHO model has increased plasma resistin compared with ApoE-/- mice as well as increased white adipose tissue-derived resistin, showing that HF feeding can bypass miR-155 deficiency-defined lower inflammation status and accelerate the transition from less inflamed MHO to highly inflamed classical obesity with our newly defined “secondary wave of inflammation status (SWIS).” These experimental data strongly consolidate our new working model on the two types of MHO proposed based on our experimental data analysis.

**FIGURE 9:**
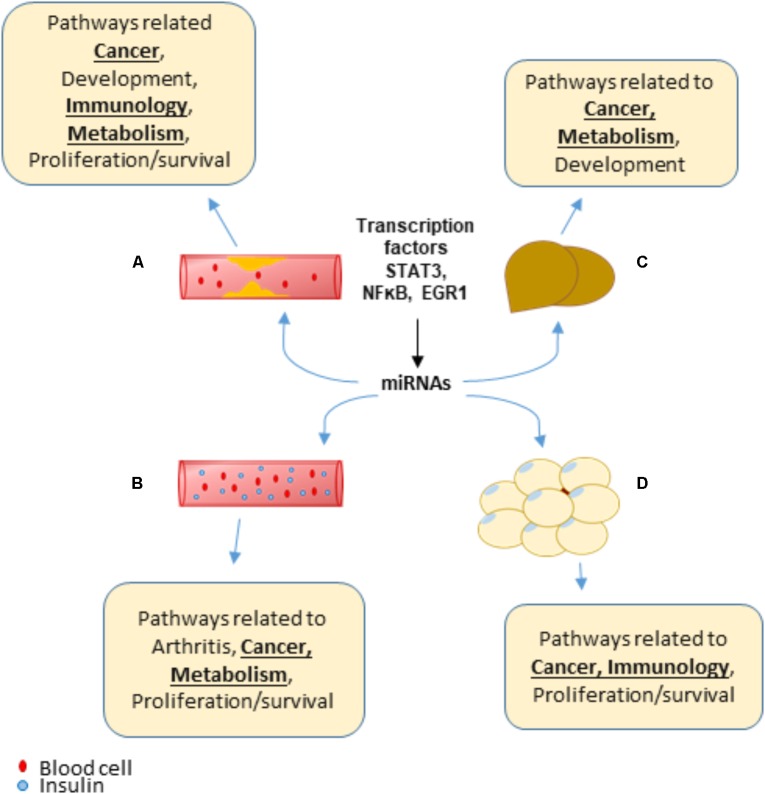
A New Working Model: Transcription factors, such as STAT3, NFκB, and EGR1, can promote the transcript levels of various miRNAs that are both shared between and unique to hyperlipidemia diseases. Over time, these miRNAs may play a role to promote pathways related to cancer, arthritis, proliferation/survival and more, which leads to additional diseases. **(A)** Atherosclerosis. **(B)** Type 2 diabetes/insulin resistance. **(C)** Non-alcoholic fatty liver disease. **(D)** Obesity.

In summary, we propose for the first time a new working model showing that transcription factors, such as STAT3, NFκB and EGR1, increase transcript levels of various miRNAs that are unique to the four HRDs (atherosclerosis, NAFLD, obesity, and T2DM/IR) as well as shared among the HRDs. These miRNAs, expressed in various tissues inhibit translation of or degrade mRNA targets that not only help give rise to HRD phenotype but may also be an underlying mechanism of MHO, cancer, arthritis and other conditions involving proliferation/survival, development, immunology, and metabolism pathways (**Figure 9**). Experimental studies would be needed to confirm these findings.

## Author Contributions

CJ carried out data gathering and data analysis, prepared the tables and the figures, and drafted the manuscript. AV aided with data analysis. CD, MH, TG, SW, and LS aided with data gathering. HW supervised the experimental design. X-FY supervised the experimental design, data analysis, table and figure preparation, and the manuscript writing.

## Conflict of Interest Statement

The authors declare that the research was conducted in the absence of any commercial or financial relationships that could be construed as a potential conflict of interest.

## Footnotes

1 http://www.regnetworkweb.org/

2 https://www.ncbi.nlm.nih.gov/gds
