# Supplementary material for: Increased Expression of Resistin in MicroRNA-155-Deficient White Adipose Tissues May Be a Possible Driver of Metabolically Healthy Obesity Transition to Classical Obesity
Source: Front Physiol. 2018 Oct 12;9:1297. doi: 10.3389/fphys.2018.01297 (PMC6194169; doi:10.3389/fphys.2018.01297)
Supplement: Supplementary file 1 [file Table_1.DOCX]

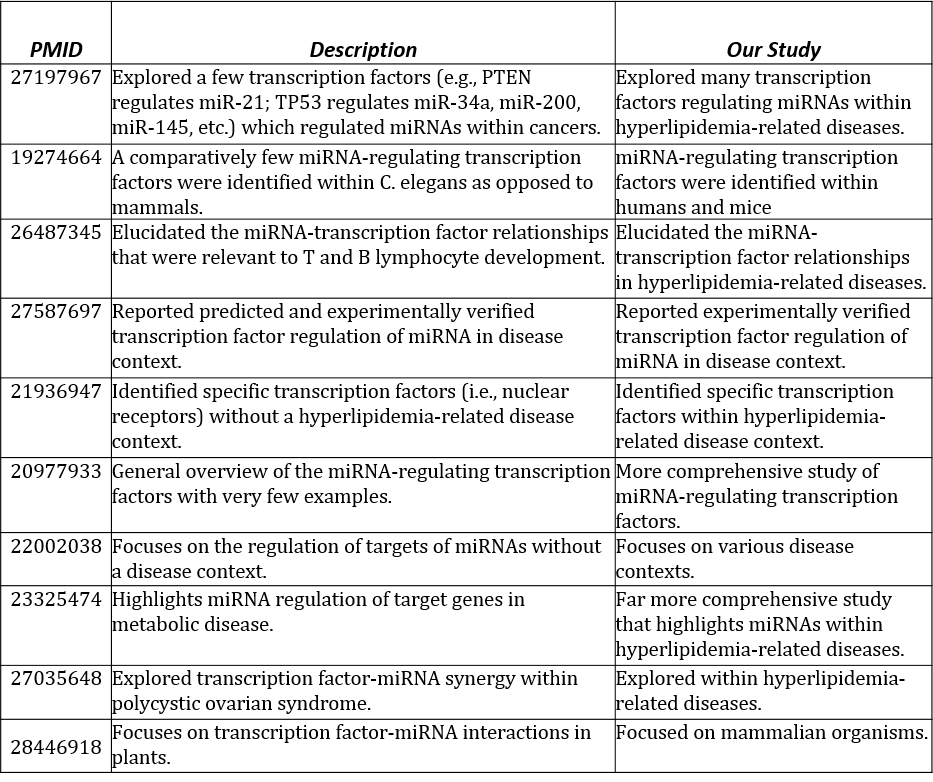


**Suppl. Table 1.** Publications that highlight miRNA and transcription factor predicted or verified interactions but none within a metabolic disease-related conditions.
