# Supplementary figures and images for: Increased Expression of Resistin in MicroRNA-155-Deficient White Adipose Tissues May Be a Possible Driver of Metabolically Healthy Obesity Transition to Classical Obesity
Source: Front Physiol. 2018 Oct 12;9:1297. doi: 10.3389/fphys.2018.01297 (PMC6194169; doi:10.3389/fphys.2018.01297)

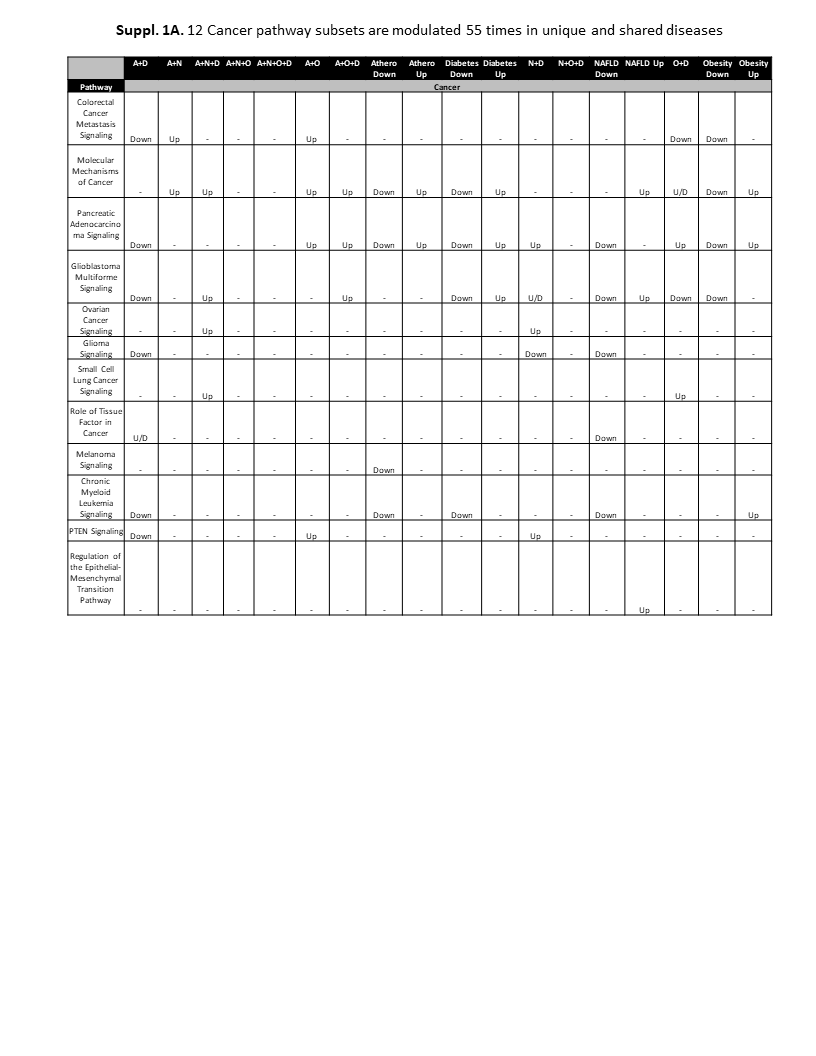

Supplement: Supplementary file 2 [file Image_1.tif]

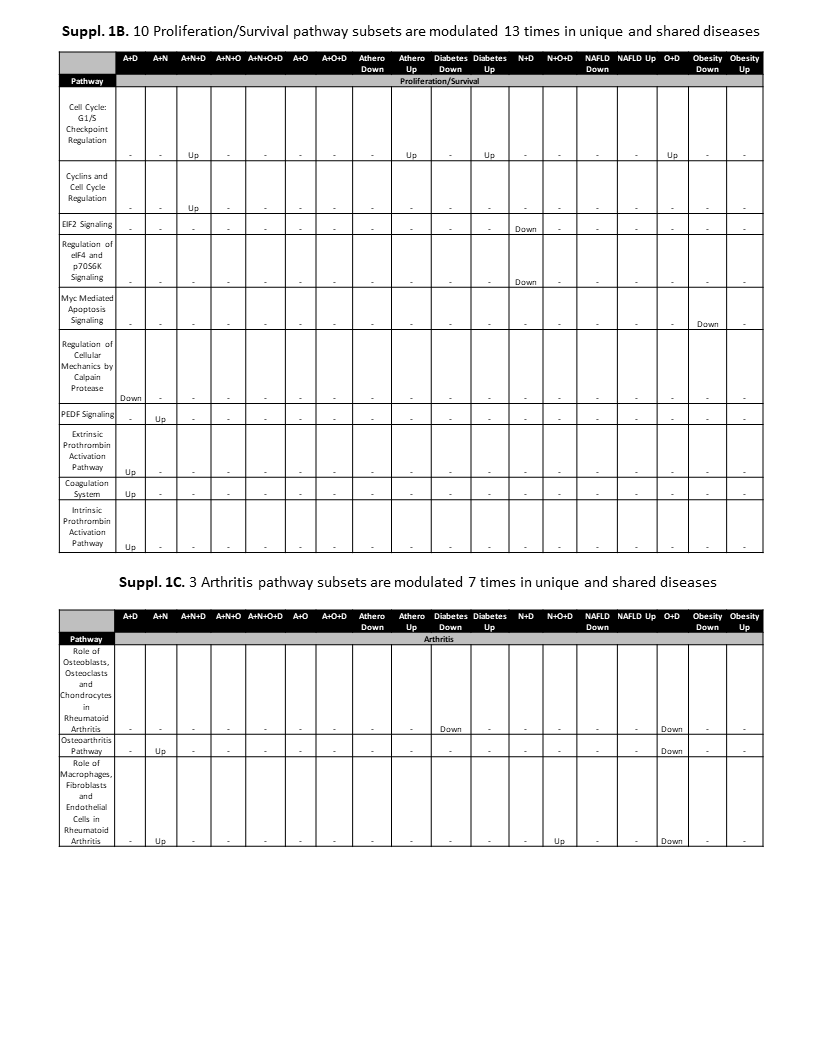

Supplement: Supplementary file 3 [file Image_2.tif]

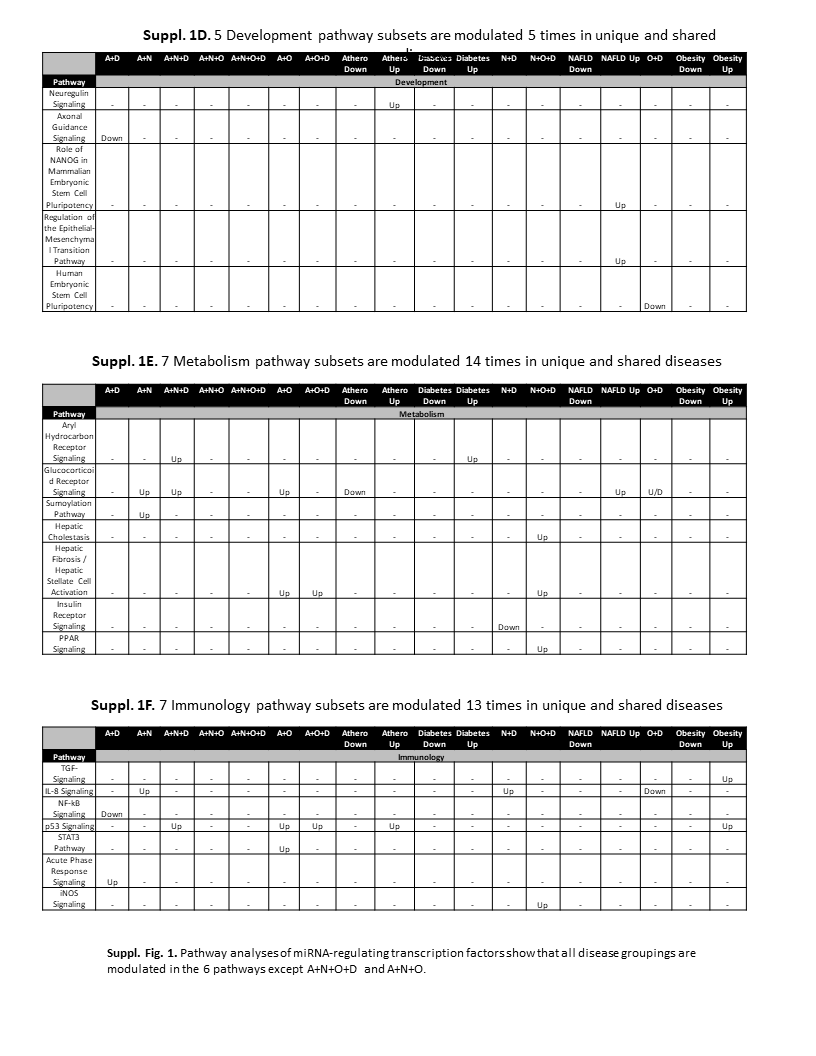

Supplement: Supplementary file 4 [file Image_3.tif]

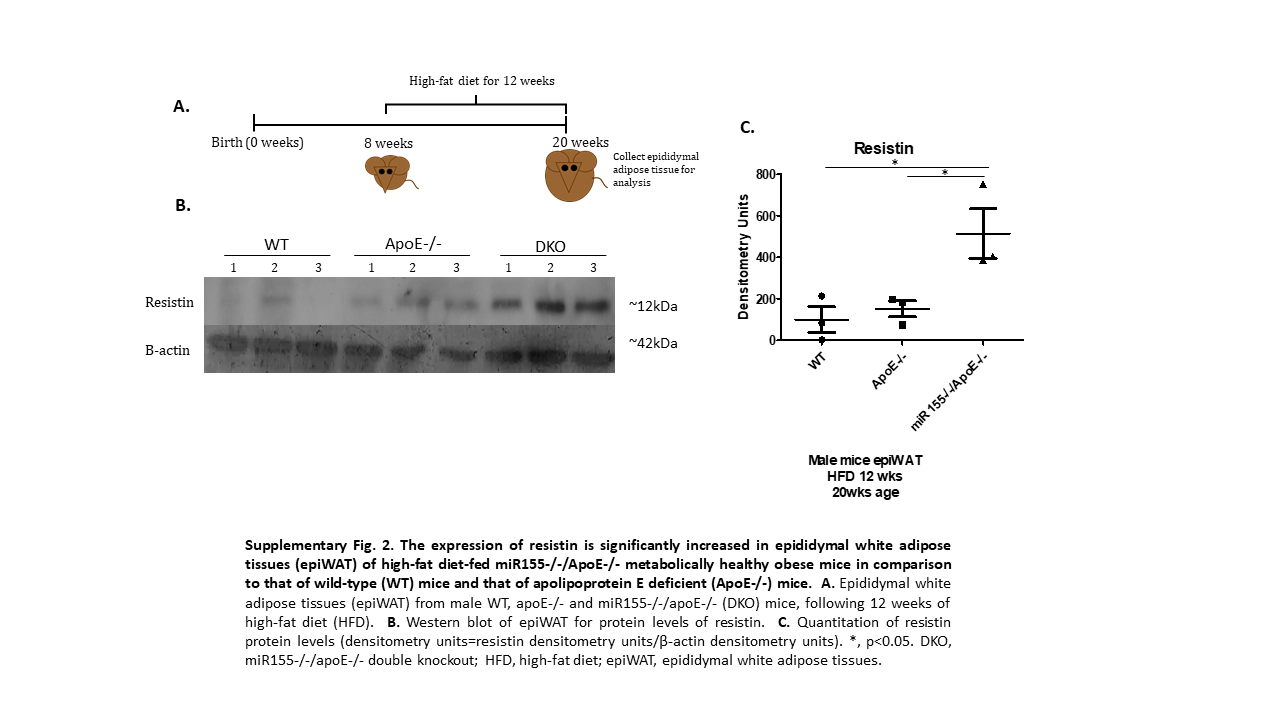

Supplement: Supplementary file 5 [file Image_4.tif]

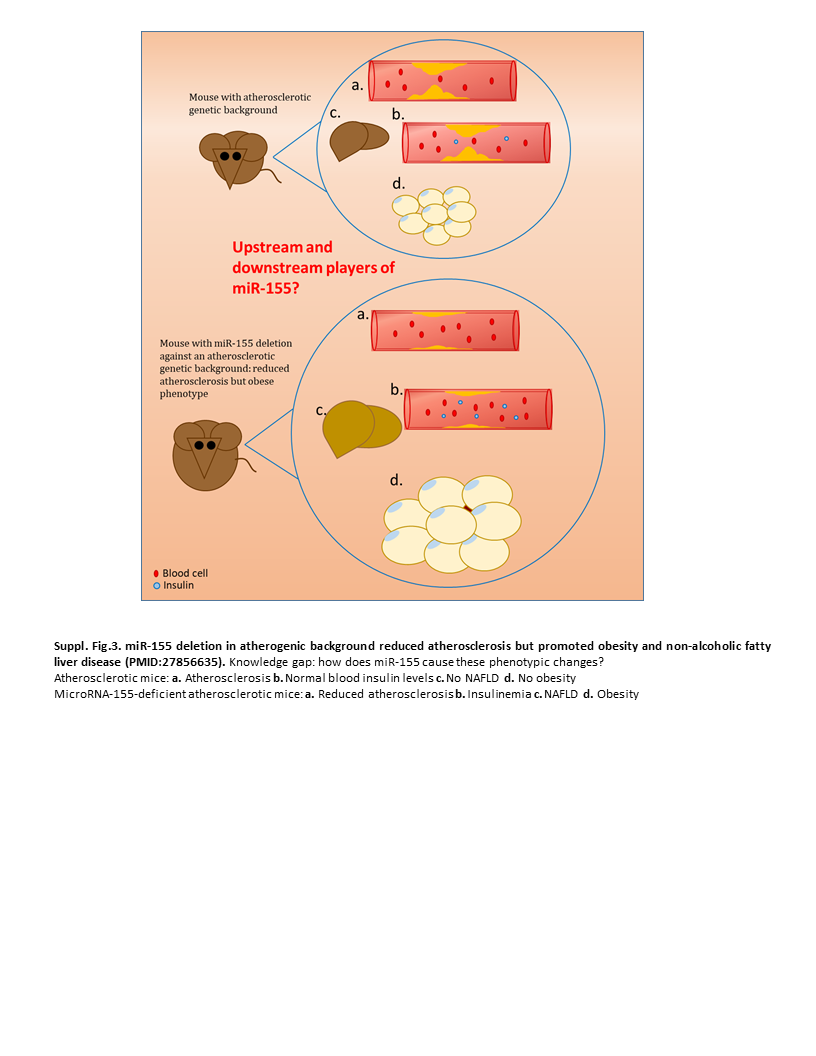

Supplement: Supplementary file 6 [file Image_5.tif]
